# Non‐target site SDHI resistance is present as standing genetic variation in field populations of Zymoseptoria tritici

**DOI:** 10.1002/ps.4761

**Published:** 2017-11-23

**Authors:** Masao Yamashita, Bart Fraaije

**Affiliations:** ^1^ Rothamsted Research, Biointeractions and Crop Protection Department Harpenden UK; ^2^ Research Centre Nihon Nohyaku Co. Ltd Osaka Japan

**Keywords:** Septoria leaf blotch, fungicide resistance, succinate dehydrogenase inhibitor, fluopyram, isofetamid

## Abstract

**BACKGROUND:**

A new generation of more active succinate dehydrogenase (Sdh) inhibitors (SDHIs) is currently widely used to control Septoria leaf blotch in northwest Europe. Detailed studies were conducted on Zymoseptoria tritici field isolates with reduced sensitivity to fluopyram and isofetamid; SDHIs which have only just or not been introduced for cereal disease control, respectively.

**RESULTS:**

Strong cross‐resistance between fluopyram and isofetamid, but not with other SDHIs, was confirmed through sensitivity tests using laboratory mutants and field isolates with and without Sdh mutations. The sensitivity profiles of most field isolates resistant to fluopyram and isofetamid were very similar to a lab mutant carrying SdhC‐A84V, but no alterations were found in SdhB, C and D. Inhibition of mitochondrial Sdh enzyme activity and control efficacy in planta for those isolates was severely impaired by fluopyram and isofetamid, but not by bixafen. Isolates with similar phenotypes were not only detected in northwest Europe but also in New Zealand before the widely use of SDHIs.

**CONCLUSION:**

This is the first report of SDHI‐specific non‐target site resistance in Z. tritici. Monitoring studies show that this resistance mechanism is present and can be selected from standing genetic variation in field populations. © 2017 The Authors. *Pest Management Science* published by John Wiley & Sons Ltd on behalf of Society of Chemical Industry.

## 1 INTRODUCTION

Fungicides are widely used in crop protection to achieve quality and a high yield of produce. Septoria leaf blotch, caused by *Zymoseptoria tritici* (synonym: *Mycosphaerella graminicola*), is one of the most important diseases affecting wheat production in northwest Europe. Owing to a lack of resistant varieties, programmed application of fungicides has been key to controlling this pathogen.^1^ Methyl benzimidazole carbamates, sterol demethylation inhibitors and quinone outside inhibitors (QoIs) have been introduced sequentially to the market and have provided growers with excellent control of Septoria leaf blotch. However, their efficacy has been lost or substantially eroded over time due to the emergence and further spread of resistant strains in *Z. tritici* field populations.^2^, ^3^, ^4^

Carboxamide fungicides, representing an old class of chemistry originating from the late 1960s, have been shown to inhibit succinate dehydrogenase (Sdh), an important component of the mitochondrial respiratory chain (complex II). Succinate dehydrogenase inhibitors (SDHIs) impact electron transport by blocking the quinone‐binding site of Sdh formed by subunits B, C and D.^5^, ^6^, ^7^, ^8^ In contrast to the narrow spectrum of early‐generation SDHIs, the latest generation of SDHIs have shown broad‐spectrum control of Ascomycota, including *Z. tritici*.^9^, ^10^ Following the 2003 introduction of boscalid, the first of the new generation of SDHIs with strong eyespot activity,^9^ other SDHIs, such as bixafen, fluxapyroxad, isopyrazam, penthiopyrad and benzovindiflupyr, that are very effective in controlling Septoria leaf blotch have also been registered in Europe since 2010. To delay resistance, SDHIs should be used in a mixture with other fungicides having different modes of action, such as azoles, and/or multiple sites, and the maximum number of sprays per season has been restricted.

Several laboratory ultraviolet (UV)‐mutagenesis studies have shown that SDHI resistance can develop easily in different fungal species,^11^, ^12^ including *Z. tritici*, for which a range of mutations in the *SdhB*, *SdhC* and *SdhD* genes have been detected.^13^, ^14^, ^15^ Prior to the emergence of SDHI resistance in *Z. tritici* field strains in 2012,^16^ SDHI resistance‐conferring mutations underlying single amino acid substitutions in Sdh subunits B, C or D were reported for field strains of other plant pathogens.^17^, ^18^, ^19^ Lack of a cross‐resistance relationship between boscalid and fluopyram has been found in several cases. Substitution of histidine by tyrosine at codon position 272 in SdhB (B‐H272Y) of *Botrytis cinerea* and an equivalent substitution in *Corynespora cassiicola* led to very high resistance to boscalid, although sensitivity to fluopyram remained.^20^ Furthermore, the new SDHI isofetamid had a sensitivity profile similar to that of fluopyram when a B‐H272Y mutant of *B. cinerea* was tested.^21^, ^22^ Low levels of SDHI resistance in *Z. tritici* strains carrying Sdh mutations C‐T79 N and C‐W80S were reported in 2012, followed by C‐N86S (2013), B‐N225 T (2014) and B‐T268I (2015).^16^ High levels of resistance due to strains carrying Sdh mutation C‐H152R were recently reported in Ireland and the UK.^16^, ^23^, ^24^ Field isolates with different levels of sensitivity to fluopyram have also been found in Ireland but not discussed further.^23^

Metabolic degradation or altered expression of efflux pumps encoded by ATP‐binding cassette (ABC) transporters and/or major facilitator superfamily (MFS) transporters can also reduce sensitivity against various xenobiotics, including fungicides with different modes of action.^25^, ^26^, ^27^, ^28^ In *Z. tritici*, upregulation of *MgMFS1* by a 519‐bp insertion in the promoter region led to a decrease in sensitivity to various fungicides, including QoI, SDHI and azole fungicides, although other transporters might also contribute to fungicide resistance.^28^ Generally, this type of reduced sensitivity, known as multidrug resistance (MDR), can be easily distinguished from target‐site resistance by low–moderate resistance to unrelated chemicals such as cycloheximide, rhodamine and fentin chloride, which are antifungal but also substrates of efflux pumps. Antimycotic drugs inhibiting squalene epoxidase such as terbinafine and tolnaftate have been reported as useful indicators to identify both *Z. tritici* and *B. cinerea* strains with the MDR phenotype because a high level of resistance for MDR strains was observed, especially with tolnaftate.^28^, ^29^, ^30^

Our aim of this study is: (i) to confirm a cross‐resistance relationship between SDHIs with similar chemical structures, (ii) to check the distribution of resistance against fluopyram and isofetamid in a population collected at different locations and over time, and (iii) to investigate the resistance mechanism. Here, we report further studies on the detection and characterization of fluopyram‐ and isofetamid‐resistant *Z. tritici* field strains isolated from different countries. Isofetamid is not commercialized as a cereal diseases control agent, but fluopyram has just been introduced in the UK in a mixture with bixafen and prothioconazole to enhance and obtain a wider spectrum of disease control. We observed a positive cross‐resistance relationship between fluopyram and isofetamid. Sequencing analysis of the SDHI binding pocket (*SdhB*, *SdhC* and *SdhD*) and mitochondrial Sdh enzyme activity assays revealed that the inhibitory effects of both fluopyram and isofetamid were severely impaired in the absence of any target‐site alterations in resistant strains. Further studies are needed to elucidate the underlying resistance mechanism(s) in these strains.

## 2 MATERIALS AND METHODS

### 2.1 Isolation and storage of Z. tritici strains

For cross‐resistance studies, reference strain IPO323‐derived laboratory mutants and field isolates, for which the *Sdh* genotypes had been analysed previously,^14^, ^16^, ^24^ were tested. For additional field population monitoring studies, sampling and isolation of *Z. tritici* strains from infected wheat leaves were performed as described previously.^31^ Septoria leaf blotch‐infected leaves were randomly collected from wheat fields located near Lyon (fungicide untreated plots) and Orleans (fungicide untreated and treated plots) in France and at Rothamsted Research (Harpenden, UK) from an untreated field in 2015. Additional populations were sampled from untreated fields near Carlow (Ireland) and at Rothamsted in 2017. Conidial suspensions were streaked onto yeast extract peptone dextrose agar (YPD agar; For Medium, Norwich, UK) amended with penicillin G sodium salt and streptomycin sulphate at 100 mg mL^−1^ respectively, and incubated for 7 days at 15 °C. Single yeast‐like forming colonies were propagated by transferring these to fresh YPD agar plates. Propagated spores were stored in 80% (v/v) glycerol at −80 °C. The SDHI‐sensitive reference strain MM20,^14^ carrying *SdhC*‐N33 T and C‐N34 T, isolated from a fungicide‐untreated field in Spain in 2006, and NT321.17, a SDHI‐resistant *MgMFS1* overexpressing strain^28^ without Sdh alterations, isolated from a SDHI‐treated plot in Hampshire (UK) in 2013, were included as additional reference strains in this study. NT321.17 showed the highest resistance to SDHIs among *MgMFS1* overexpressing strains in the authors' collection. In addition, strains representing field populations from the UK, Ireland and New Zealand, sampled previously and stored in the same way, were also tested.

### 2.2 Fungicides

Analytical grade compounds, including SDHIs (bixafen, boscalid, fluopyram, fluxapyroxad and penthiopyrad), fentin chloride, chlorothalonil and tolnaftate, were all purchased from Sigma‐Aldrich (UK). Isofetamid (*N*‐[1,1‐dimethyl‐2‐(4‐isopropoxy‐*o*‐tolyl)‐2‐oxoethyl]‐3‐methylthiophene‐2‐carboxamide) was synthesized and supplied with >95% purity by Nihon Nohyaku Co. Ltd. (Japan). Chemicals were dissolved in pure dimethyl sulfoxide (DMSO) at 10 mg mL^−1^ and stored at −20 °C before further use.

### 2.3 Fungicide sensitivity testing

Sensitivity tests were conducted according to Fraaije *et al*.^14^ Flat‐bottomed 96‐well microtiter plates (Greiner Bio‐One Ltd, Stonehouse, UK) were filled with 100 μL of double‐strength Sabouraud dextrose (SAB) liquid medium without or amended with different concentration of the test fungicides. The following fungicide concentrations were used: fentin chloride and chlorothalonil, 0.0098, 0.0195, 0.039, 0.078, 0.156, 0.313, 0.625, 1.25, 2.5, 5 and 10 mg L^−1^; tolnaftate, a single dose of 10 mg L^−1^ or 0.0195, 0.039, 0.078, 0.156, 0.313, 0.625, 1.25, 2.5, 5, 10 and 20 mg L^−1^; and for the SDHIs, 0.0002, 0.0009, 0.0037, 0.0146, 0.0586, 0.234, 0.938, 1.875, 3.75, 7.5 and 15 mg L^−1^ for sensitive and moderate resistant strains and 0.0052, 0.0131, 0.0328, 0.0819, 0.205, 0.512, 1.28, 3.2, 8, 20 and 50 mg L^−1^ for resistant strains. Spore suspensions of *Z. tritici* were prepared at the final concentration of 2.5 × 10^4^ spores mL^−1^ from cultures grown for 7 days on YPD agar at 15 °C. Aliquots of 100 μL of these spore suspensions were added to each well. Plates were incubated in the dark at 21 °C for 4 days, and growth was measured at 630 nm using a FLUOstar OPTIMA microplate reader (BMG Labtech, Offenburg, Germany) in well‐scanning mode with a 2 × 2 matrix of scanning points within a 3‐mm diameter. Fungicide sensitivities were determined as the 50% effective concentration to inhibit growth (EC_50_ in mg mL^−1^) using a dose–response relationship curve function of the OPTIMA software. No adverse effect of DMSO up to 500 mg mL^−1^ was observed.

### 2.4 Isolation of genomic DNA, PCR detection of MgMFS1 promoter inserts and sequencing of sdhA, B, C and D genes

Genomic DNA was extracted from strains grown on YPD plates at 15 °C in the dark for 7 days and quantified according Rudd *et al*.^32^ PCRs using primers listed in Table 1 were carried out on a Biometra T3000 thermocycler (Göttingen, Germany) in a final volume of 25 μL containing 20 ng of fungal template DNA. PCRs for amplification of *SdhB* or *SdhD* contained 0.5 μM for each primer and 150 μM dNTP, 1× Phusion HF buffer and 0.5 units of Phusion High Fidelity DNA polymerase (New England Biolabs, Ipswich, MA, USA). Amplification conditions were 98 °C for 30 s, followed by 40 cycles at 98 °C for 10 s, 57 °C (*SdhD*) or 63 °C (*SdhB*) for 30 s and 72 °C for 1 min with a final DNA extension at 72 °C for 5 min. PCRs for amplification of *SdhA, SdhC* and the *MgMFS1* promoter region contained 0.5 μM for each primer and 200 μM dNTP, 1× of Easy‐A reaction buffer and 1.25 units of Easy‐A High Fidelity PCR Cloning Enzyme (Agilent Technologies, Cedar Creek, TX, USA). Amplification conditions were 95 °C for 2 min, followed by 40 cycles at 95 °C for 10 s, 57 °C (*MgMFS1*) or 62 °C (*SdhA*) for 20 s and 72 °C for 1 min with a final DNA extension at 72 °C for 5 min. PCR products were sequenced by MWG Eurofins Genomics GmbH (Ebersberg, Germany) using both PCR primers for each reaction and two additional primers, SdhAF2 (TCTTTGCCATTGATCTCATCATG) and SdhAR2 (GCTCCGTAGATACCAGTTGGGT) were used for *SdhA* sequencing. Sequences were assembled and aligned with Geneious version 6.1.4 software (Biomatters Ltd., Auckland, New Zealand), and amino acid substitutions deduced after sequence analysis.

**Table 1:** Primers used to amplify Sdh genes and MgMFS1 promoter inserts

Primers and sequences (5′–3′)^a^	Target	Size^b^ (bp)
SdhAF1: CTGAACCTCTCCACCATCGAC	*SdhA*	2077
SdhAR1: CGGCTCTACAATTCTGGGAGAC		
SdhBF1: TAAACACTCCACGCCTCACG	*SdhB*	1270
SdhBR1: GTCTTCCGTCGATTTCGAGAC		
SdhCF1: CTACAARAAMGCCAAMCCCAAC	*SdhC*	749
SdhCR1: ATGTTGGCACAGAAGCTCAC		
SdhDF1: CGGGAATAACCAACCTCACT	*SdhD*	840
SdhDR1: CCTCACTCCTCCAAACCGTA		
MFF1: AAGGTAGGTGAACACCTTATACTC	*MgMFS1* promoter	490 or 1009
MFR1: TTCTTGCTGAAGAAGCGCATGGTTGT		

a SdhBF1 primer sequence according Dubos *et al*.,^38^ SdhDF1 and SdhDR1 primer sequences reported by Dooley *et al*.^23^

b Slight differences in amplicon sizes can be obtained due to size difference of introns of Sdh genes or variation in the *MgMFS1* promoter insert length.

### 2.5 Mitochondrial isolation and succinate dehydrogenase enzyme activity assays

Mitochondrial extraction was performed using the method by Scalliet *et al*. with minor modification.^15^ Frozen spores were homogenized in liquid nitrogen and crushed to a fine powder using a pestle and mortar; 20% w/v of the powder was re‐suspended in mitochondrial extraction buffer (1 M sorbitol, 50 mM sodium citrate buffer, pH 5.8) containing 1 mM dithiothreitol. The suspension was centrifuged at 700 *g* for 5 min once and supernatant was transferred to new tubes. The supernatant was then centrifuged and mitochondria were pelleted at 17000 *g* for 20 min. A pink–red pellet was re‐suspended in assay buffer (0.25 M sucrose, 0.1 mM EDTA, 3 mM Tris–HCl, pH 7.4) and washed by centrifugation. After measuring the protein content using the Bradford protein assay,^33^ isolated mitochondria samples were adjusted to a final concentration of 5 μg protein μL^−1^ and immediately used in enzyme assays. Colorimetric assay using microplate reader was adapted to measure succinate: ubiquinone/dichlorophenolindophenol (DCIP) activity. Briefly, 2 μg protein samples of isolated mitochondria were added to 100 μL of assay buffer containing 200 μM 2,3‐decyl ubiquinone (dUQ) and SDHI solution at different concentrations (0, 0.002, 0.008, 0.031, 0.125, 0.5, 2, 8 and 32 μM). Then, 100 μL of reaction buffer (assay buffer containing 100 μM DCIP, 10 mM succinate and 1 μM antimycin A) was added to initiate the enzyme reaction and the reduction in DCIP was monitored at 600 nm using a iMark microplate reader (Bio‐Rad Laboratories, Hercules, CA, USA).

### 2.6 In planta Septoria efficacy testing using different SDHIs

Wheat cultivar, Consort, was sown on commercial nursery soil. Four‐week‐old seedlings, four per pot, in triplicate, were sprayed with fungicide solution prepared with an in‐house emulsifiable concentrate (EC) formulation containing surfactant and organic solvent, dried for several hours and inoculated with a spore suspension of *Z. tritici* at a concentration of 2 × 10^6^ spores mL^−1^. Because IPO323 was not able to infect cv Consort, strain MM20 was used as reference for efficacy tests.^14^ After 48 h at 100% relative humidity (RH) in dark incubation boxes, seedlings were moved to the greenhouse and incubated for ∼ 18 days at >80% RH and ambient temperature. Disease symptoms were assessed visually using the following keys: 0, no symptoms; 1, ∼ 10%; 2, 10–25%; 3, 25–50%; 4, 50–80%; and 5, > 80% of leaf area covered with pycnidia. Control efficacy was calculated using the following formula:

Control efficacy (%) = 100 × (1 – average of key values in fungicide‐treated plot/ average of key values in untreated plot).

Final data sets were based on the average of each test performed three times independently.

### 2.7 Statistical analysis

All statistical analysis was performed by SAS (SAS Institute Inc., Cary, NC, USA). For cross‐resistance between SDHIs, Spearman's correlation analysis was applied to EC_50_ values of SDHIs against each *Sdh* mutants. The Shapiro–Wilk test was used to assess normality for field population collected in 2015.

## 3 RESULTS

### 3.1 SDHI cross‐resistance patterns in resistant lab mutants and field strains

**Figure 1:**
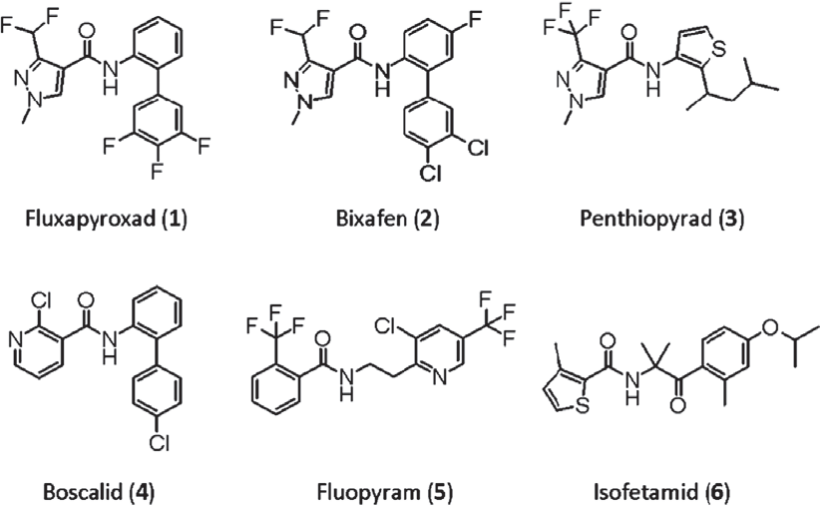
Chemical structures of six SDHIs tested in this study. Fluxapyroxad (1), bixafen (2), penthiopyrad (3), boscalid (4), fluopyram (5) and isofetamid (6).

**Figure 2:**
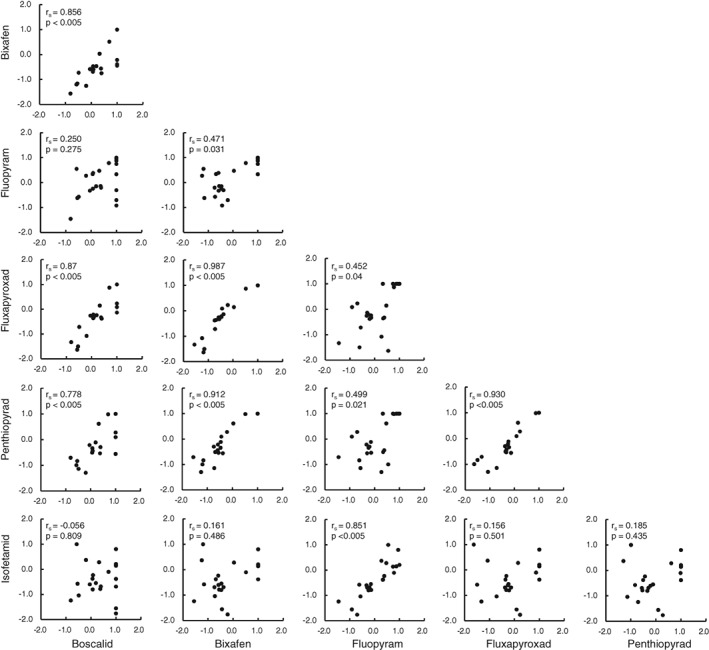
Spearman's correlations between six SDHIs for Zymoseptoria tritici isolates with Sdh mutations shown in Table 2 (n = 22). Sensitivity data measured as EC_50_ (mg L^−1^) values were expressed with log_10_ scale. P < 0.05 means the correlation was statistically significant.

In total, six different SDHIs belonging to four chemical groups were tested (Fig. 1). To assess SDHI cross‐resistance patterns, a range of laboratory‐generated UV mutants and two 2015 UK field isolates carrying C‐T79 N and C‐I161S, which have been reported previously, were tested (Table 2).^14 − 16,24^ The number of data points (*n* = 21) was not enough; however, a positive cross‐resistance relationship was observed between the pyridine (boscalid) and pyrazole carboxamides (bixafen, fluxapyroxad and penthiopyrad), with values of *r_s_* > 0.778 (Fig. 2). The pyridinyl‐ethyl‐benzamide fluopyram and the phenyl‐oxo‐ethyl thiophene amide isofetamid had slightly higher fungicidal activity against the B‐H267Y mutants (18 − 11 and M36) compared with reference strain IPO323, and showed a high level of cross‐resistance (*r_s_* = 0.851), whereas weaker correlations between the other four SDHIs were observed with *r_s_* values ranging from 0.250 to 0.499 for fluopyram and −0.056 to 0.185 for isofetamid, respectively. Fungicidal activities of both fluopyram and isofetamid were less impaired by mutants for whom the other four SDHIs were severely affected, such as B‐T268I and C‐T79I. However, in comparison with the other SDHIs, the C‐A84V mutant was much less sensitive to isofetamid and fluopyram with resistance factors (RF) of >174 and >18, respectively.

**Table 2:** Sensitivity profiles of laboratory‐generated Sdh mutants and filed isolates of Z. tritici against six SDHIs

Isolate	Mutation in Sdh subunit	Amino acid substitution		Resistance factor (RF)^a^					
			Origin^b^	Boscalid	Bixafen	Fluxapyroxad	Penthiopyrad	Fluopyram	Isofetamid
M38	B	D129T	lab	32.3	118.1	211.2	206.4	30.8	13.7
Iso − 13	B	P220T	lab	4.1	2.0	2.4	1.1	9.6	40.7
M152	B	S221P	lab	1.9	2.5	0.9	3.1	1.2	4.6
M6	B	R265P	lab	15.2	10.0	13.3	6.2	3.7	2.9
M46	B	R265P	lab	15.9	6.5	11.9	10.8	3.2	3.6
18 − 11	B	H267Y	lab	> 64	13.0	34.6	26.5	0.6	0.5
M36	B	H267Y	lab	> 64	22.0	48.0	40.3	1.0	0.3
15‐8	B	H267L	lab	> 64	> 363	> 284	> 213	45.3	110.2
V5 − 1	B	T268I	lab	5.7	9.5	15.7	13.0	2.4	4.4
V5 − 12	B	T268I	lab	7.5	12.2	17.2	9.8	2.9	2.8
M62	B	I269V	lab	7.4	7.4	12.4	6.6	11.2	7.3
M96	B	I269V	lab	7.7	9.3	13.4	7.8	12.4	10.1
20 − 13	C	T79I	lab	> 64	> 363	> 284	> 213	11.0	7.2
16 − 12	C	S83G	lab	> 64	> 363	207.7	> 213	> 51	28.1
Flu‐6	C	A84V	lab	1.7	2.3	0.7	2.2	18.1	> 174
Iso‐30	C	L85P	lab	13.5	39.1	39.8	87.9	15.1	33.1
M142	C	N86 K	lab	> 64	> 363	> 284	> 213	38.2	24.6
12 − 17	C	H152R	lab	> 64	> 363	> 284	> 213	28.3	24.0
V9C‐23	C	I161S	field	2.1	6.8	5.4	1.5	1.4	1.6
V6A‐9	C	T79 N, V128 M	field	10.1	12.4	16.3	16.4	3.6	4.9
M112	D	D129E	lab	> 64	14.9	20.8	5.9	2.5	3.6
IPO323 (EC_50_; mg L^−1^)		none	field	0.16	0.03	0.04	0.05	0.20	0.06

a Ratio between EC_50_ value of each isolate and of IPO323. Owing to dose–response curve‐fitting and/or solubility, 10 mg L^−1^ was used as the cut‐off value for EC_50_ determination. Each value is based on the means of two individual EC_50_ values.

b IPO323‐derived laboratory mutant (lab) or UK field isolates collected in Norfolk (V6A‐9) and Wiltshire (V9C‐23) in 2015.

### 3.2 SDHI sensitivity testing and SdhB, C and D sequence analysis of Z. tritici strains isolated in France and the UK in 2015

**Figure 3:**
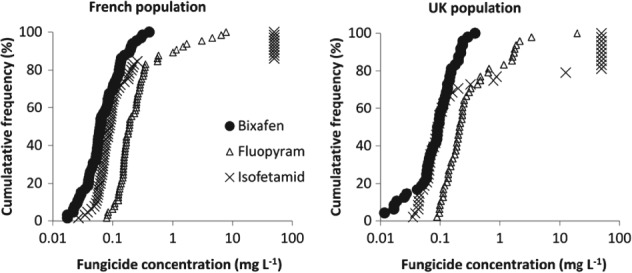
Sensitivity distribution of French and UK strains of Zymoseptoria tritici to bixafen, fluopyram and isofetamid. Isolates are ranked according increasing EC_50_ values (cumulative). French strains (n = 65) sampled from untreated plots near Lyon (n = 33) and Orleans (n = 32) in 2015. UK strains (n = 48) sampled from untreated plots at Rothamsted Research (Harpenden) in 2015.

The bixafen, fluopyram and isofetamid sensitivity profiles of 113 single‐spore isolates collected from untreated plots in France (strains from Lyon and Orleans; *n* = 65) and the UK (strains from Harpenden; *n* = 48) were measured (Fig. 3 and Table 3). The EC_50_ values of bixafen ranged from 0.017 to 0.406 mg L^−1^ and from 0.011 to 0.38 mg L^−1^ for French and UK isolates, respectively, and were normally distributed (*P* = 0.61 and 0.06). However, the EC_50_ sensitivity for both fluopyram and isofetamid was much wider due to high EC_50_ values for fluopyram (up to 7.78 and 19.51 mg L^−1^ for strains from France and the UK, respectively) and isofetamid (> 50 mg L^−1^ for both populations), and not normally distributed (*P* < 0.005).

**Table 3:** SDHI sensitivity ranges and distributions for French and UK strains isolated in 2015

Active ingredient	France			UK		
	EC_50_ (mg L^−1^)		W‐test^a^	EC_50_ (mg L^−1^)		W‐test^a^
	Range	Median		Range	Median	
Bixafen	0.017 to 0.406	0.063	*P* = 0.61	0.011 to 0.38	0.096	*P* = 0.06
Fluopyram	0.080 to 7.784	0.190	*P* < 0.005	0.089 to 19.51	0.215	*P* < 0.005
Isofetamid	0.027 to >50	0.090	*P* < 0.005	0.035 to >50	0.096	*P* < 0.005

a Shapiro–Wilk test for normal distribution (log_10_ scale); *P* < 0.05 assumed to be not normally distributed.

**Figure 4:**
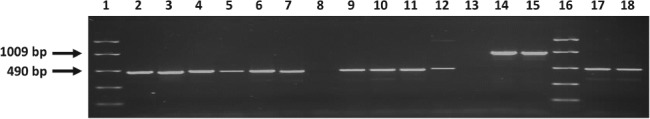
PCR detection of the 519‐bp MgMFS1 promoter insert in Zymoseptoria tritici strains using primers MFF1 and MFR1. Samples of EasyLadder I (Bioline, London, UK) in lanes 1 and 16, products of strains Orleans 26 (lane 2), Lyon 35 (3), R15‐46 (4), Orleans 8 (5), Lyon 26 (6), Orleans 6 (7), Lyon 24 (8), Orleans 12 (9), Orleans 11 (10), Lyon 14 (11), Orleans 40 (12), Lyon 31 (13), Lyon 16 (14), NT321.17 (15), IPO323 (17) and Flu‐6 (18). No amplification for Lyon 24, Lyon 31 and water control (not shown). Largest product (1009 bp) in lane 14 and 15, Lyon 16 and NT321.17, respectively, indicates the presence of 519 bp promoter insert; no insert present in smaller 490 bp product.

Thirteen 2015 field isolates, identified as either sensitive (*n* = 3) or resistant to isofetamid (*n* = 10), were selected and further characterized using SDHI sensitivity testing and *SdhB*, *C* and *D* sequence analysis (Table 4). Sensitivity testing confirmed the high levels of resistance to isofetamid in the resistant strains with RF > 174 in comparison with reference strain IPO323. The level of fluopyram sensitivity varied in the isofetamid‐resistant strains, with RF ranging from 1.2 to 38.6, but high values (RF > 10) were measured for six of the 10 strains tested. Furthermore, none of these strains was able to grow in the presence of 10 mg L^−1^ tolnaftate and no or a low level of resistance (RF < 10) was measured for all compounds tested, including the SDHIs fluxapyroxad and bixafen. The sensitivity levels of the three isofetamid sensitive strains, Orleans 40, Lyon 31 and Lyon 16, were as expected; no or low levels of sensitivity to all compounds with exception of strain Lyon 16, in which low levels of resistance were measured for all SDHIs and fentin chloride, together with a high level of resistance to tolnaftate (RF > 58). A similar pattern, albeit with higher RF values, was found for the efflux pump *MgMFS1* overexpressing reference strain NT321.17. PCR confirmed the presence of a 519‐bp insert in the *MgMFS1* promoter region of strain Lyon 16 and reference strain NT321.17, but not in the other strains tested (Fig. 4). All strains tested, including the reference strains, were sensitive to the multisite inhibitor chlorothalonil.

**Table 4:** Sdh variants and fungicide sensitivity profiles of selected field isolates and control strains

	Resistance factor (RF)^b^							Sdh polymorphism		
	Fluopyram	Isofetamid	Bixafen	Fluxapyroxad	Chlorothalonil	Fentin Cl	Tolnaftate	Sdh B	Sdh C	Sdh D
Orleans 26	38.6	> 174	9.1	2.5	1.3	0.9	1.7	None	N33 T, N34 T	None
Lyon 35	34.0	> 174	5.6	3.1	1.3	1.0	1.5	None	N33 T, N34 T	None
R15 ‐ 46	30.5	> 174	3.1	NT^c^	NT	1.1	NT	None	N33 T, N34 T	V106 L
Orleans 8	23.3	> 174	3.8	2.4	0.7	1.0	1.5	None	None	None
Lyon 26	15.2	> 174	4.1	NT	NT	NT	NT	None	None	None
Orleans 6	10.2	> 174	1.6	NT	NT	NT	NT	none	N33 T, N34 T	None
Lyon 24	9.8	> 174	3.3	2.0	NT	NT	NT	none	None	None
Orleans 12	8.9	> 174	4.0	NT	0.8	0.8	1.2	None	None	None
Orleans 11	4.1	> 174	1.6	NT	NT	NT	NT	None	None	None
Lyon 14	1.2	> 174	1.2	2.7	NT	NT	NT	None	None	None
Orleans 40	0.9	1.1	2.8	2.9	1.2	0.5	1.1	None	None	None
Lyon 31	0.8	1.6	2.5	2.9	1.3	0.9	1.2	None	N33 T, N34 T	None
Lyon 16	2.9	4.3	13.1	NT	1.3	6.6	> 58	None	N33 T, N34 T	None
NT 321.17	19.4	29.4	64.4	45.6	1.4	11.6	> 58	None	None	None
IPO323 (EC50; mg L^−1^)^a^	0.20	0.06	0.03	0.04	0.13	0.07	0.17	None	None	None

a Values are the means of two independent EC_50_ determinations. Due to dose response curve‐fitting and/or solubility 10 mg L^−1^ was used as cut‐off value for EC_50_ determination.

b Ratio between EC_50_ value of each isolates and reference IPO323.

c Not tested.

*SdhB*, *C* and *D* sequencing analysis of all selected strains showed the presence of three different Sdh amino acid substitutions. Six of the 13 isolates tested showed two SdhC amino acid substitutions, C‐N33 T and C‐N34 T, simultaneously, but the presence of these alterations was not linked with SDHI resistance because Lyon 31 carrying those two mutations was sensitive to four SDHIs tested (Table 4). An additional substitution, D‐V106 L, was found in strain R15‐46, but this alteration is not known to affect SDHI binding,^14^, ^15^ and the bixafen sensitivity of this strain was like the other tolnaftate‐sensitive strains carrying both C‐N33 T and C‐N34 T.

### 3.3 Mitochondrial succinate dehydrogenase activity assays

Extracted mitochondria from isolates sensitive and less‐sensitive to fluopyram and isofetamid were subjected to Sdh enzyme activity assays (Table 5). The mitochondrial Sdh activity of reference strain IPO323 was severely affected with IC_50_ values of 0.033, 0.120 and 0.050 μM for bixafen, fluopyram and isofetamid, respectively. In comparison with both IPO323 and Lyon 14, a moderate fluopyram‐resistant strain (Tables 4 and 5), much higher IC_50_ and corresponding RF values were measured for fluopyram and isofetamid for the highly resistant strains Lyon 35, Orleans 26 and R15‐46. Interestingly, a similar phenotypic mitochondrial response was observed for the IPO323‐based lab mutant Flu‐6 carrying C‐A84V (Table 2) with IC_50_ values of 0.106, 14.76 and >32 μM for bixafen, fluopyram and isofetamid, respectively. However, in comparison with the all other strains tested, the IC_50_ value for bixafen was at least twofold higher for this mutant.

**Table 5:** Inhibition of mitochondrial succinate dehydrogenase (SDH) activity by different SDHIs in field isolates of Z. tritici shown to be less sensitive to both fluopyram and isofetamid

	IC_50_ (μM)			RF^b^		
	Bixafen	Fluopyram	Isofetamid	Bixafen	Fluopyram	Isofetamid
Lyon 35	0.038 ± 0.011^a^	9.672 ± 3.793	> 32	1.2	80.4	> 640
Orleans 26	0.031 ± 0.01	3.603 ± 0.994	> 32	0.9	30.0	> 640
R15‐46	0.044 ± 0.011	12.41 ± 1.322	> 32	1.3	103.1	> 640
Lyon 14	0.049 ± 0.013	1.053 ± 0.094	0.827 ± 0.018	1.4	8.3	16.7
Flu‐6	0.106 ± 0.017	14.76 ± 1.317	> 32	3.2	122.7	> 640
MM20	0.031 ± 0.017	0.134 ± 0.067	0.046 ± 0.009	0.9	1.1	0.9
IPO323	0.033 ± 0.017	0.12 ± 0.008	0.05 ± 0.002	1.0	1.0	1.0

a Values ± SD are indicated as means of three independent experiments.

b Values are indicated as ratio between means of IC_50_ (field isolates) and IC_50_ (IPO323).

### 3.4 In planta disease control of SDHI resistant Z. tritici strains

The SDHI‐sensitive reference strain MM20 and four field isolates, moderate (Lyon 14) or highly resistant to fluopyram and isofetamid (Lyon 35, Orleans 26 and R15‐46) were tested in the greenhouse (Table 6). After being inoculated on wheat seedlings, sprayed preventatively with three SDHIs to evaluate pathogenicity and *in planta* disease control, all five strains produced pycnidia on unsprayed and inoculated leaves 18 days after inoculation. MM20 was well controlled (efficacy >80%) by both fluopyram and isofetamid at dose rates of ≥10 mg L^−1^. By contrast, the three highly resistant isolates, Lyon 35, Orleans 26 and R15‐46, were not controlled by fluopyram and isofetamid even at the highest application rate of 100 mg L^−1^. The moderate resistant strain Lyon 14 was not controlled using isofetamid but fluopyram provided control at rates ≥30 mg L^−1^. Bixafen showed a high control efficacy at 1 mg L^−1^ for MM20 (91 %) and the other strains were well controlled at either 3 (Orleans 26) or 10 mg L^−1^ (Lyon 14, Lyon 35 and R15‐46).

**Table 6:** In planta control of Z. tritici strains using three different SDHIs

	Application	Control efficacy^b^				
	dose^a^	MM20	Lyon 35	Orleans 26	R 15‐46	Lyon 14
Fluopyram	100	100	13	71	0	100
	30	100	0	0	0	100
	10	98	0	0	0	22
	3	66	0	0	0	0
	1	35	0	0	0	0
Isofetamid	100	100	42	0	0	47
	30	100	35	0	0	0
	10	87	29	0	0	0
	3	68	0	0	0	0
	1	31	0	0	0	0
Bixafen	10	100	97	100	100	100
	3	100	51	96	8	77
	1	91	31	0	0	22
Untreated check^c^	—	10	10	10	10	10

a Values in mg L^−1^.

b Values are indicated as means of three independent experiments.

c Means of disease severity (0 − 10) based on area with pycnidia 18 days after inoculation.

### 3.5 Distribution of fluopyram and isofetamid resistant strains in Z. tritici populations sampled at different locations and over time

Population sensitivity profiles to three SDHIs, bixafen, fluopyram and isofetamid, were determined for 12 different field populations sampled in the UK (6), Ireland (2), France (2) and New Zealand (2) (Table 7). The frequencies of isolates with low and high resistance to fluopyram, isofetamid and bixafen in each population were determined. Highly isofetamid‐resistant strains (EC_50_ > 5.0 mg L^−1^) were detected in each population, with frequencies between 2.6% and 33.3%. Highly fluopyram‐resistant strains (EC_50_ > 5.0 mg L^−1^) were detected only at low frequencies, between 2.2% and 11.1%, in populations sampled from 2010 onwards. The frequency of fluopyram‐ and isofetamid‐resistant strains increased over time for populations sampled at Harpenden (UK) and Carlow (Ireland). No highly bixafen‐resistant strains (EC_50_ > 3.0 mg L^−1^) were detected, but low‐resistance strains (EC_50_ values between 0.3 and 3.0 mg L^−1^) were detected in French, UK and Irish populations sampled in 2015 and 2017. The high frequency of low bixafen‐resistance strains in Harpenden (19.4%) and Carlow (76.1 %) seemed to be associated with low levels of fluopyram resistance (EC_50_ values between 0.5 and 5.0 mg L^−1^) at these two locations with frequencies of 50.0% and 80.4%, respectively.

**Table 7:** Frequency of SDHI‐resistant strains in Z. tritici field populations. Frequencies (%) of low and highly resistant fluopyram, isofetamid and bixafen strains within populations are presented

Location^a^	Year	*n*	EC_50_ (mg L^−1^)					
			Fluopyram		Isofetamid		Bixafen	
			> 0.5	> 5.0	> 0.5	> 5.0	> 0.3	> 3.0
Harpenden (UK)	2003	27	3.7	0	7.4	7.4	0	0
	2010	39	7.7	2.6	15.4	12.8	NT	NT
	2015	46	23.9	2.2	28.3	23.9	2.2	0
	2017	36	50	11.1	33.3	30.6	19.4	0
Carlow (Ireland)	2003	42	7.1	0	7.1	7.1	0	0
	2017	46	80.4	4.3	45.6	15.2	76.1	0
Middlesborough (UK)	2003	38	2.6	0	2.6	2.6	0	0
Long Ashton (UK)	2003	39	10.3	0	10.3	7.7	0	0
New Zealand	2004	39	20.5	0	23.1	20.5	0	0
New Plymouth (NZ)	2008	24	41.7	0	41.7	33.3	0	0
Lyon (France)	2015	33	15.2	6.1	12.1	12.1	3	0
Orleans (France)	2015	32	18.8	3.1	18.8	18.8	3.1	0

a Populations were sampled from untreated crops at the same location with exception of the 2004 New Zealand population that contains strains sampled from one location in the North Island and four different locations on the South Island.

## 4 DISCUSSION

### 4.1 SDHI cross‐resistance studies of lab mutants and field strains of Z. tritici

Positive cross‐resistance relationships between boscalid, penthiopyrad and isopyrazam, and a lack of cross‐resistance between fluopyram and other SDHIs have been reported for SDHI‐resistant lab mutants and field strains of several fungi. Strains of *Z. tritici* carrying B‐H267Y, which is equivalent to *B. cinerea* (B‐H272Y), *A. alternata* (B‐H277Y) and *C. cassiicola* (B‐H278Y)^34^, were shown to be less sensitive to both pyridine‐ (boscalid) and pyrazole‐carboxamides (e.g. bixafen, fluxapyroxad and penthiopyrad), whereas their sensitivity to fluopyram was equal to or higher than wild‐type isolates.^15^, ^18^, ^20^, ^35^ The fungicidal activity of isofetamid was also higher for a B‐H272Y mutant of *B. cinerea*.^22^

Homology modelling and docking studies have suggested that the histidine residue at codon 267 of *SdhB* in *Z. tritici* is supposed to interact with the hetero atom, such as N and O, of the heterocyclic acid part of SDHIs via hydrogen bonding.^14^, ^36^, ^37^ The enhanced or high isofetamid and fluopyram sensitivity of two *Z. tritici* mutants carrying B‐H267Y in this study can be explained by greater hydrophobic interaction between tyrosine and these two SDHIs. An opposite trend was observed for the *Z. tritici* mutant carrying C‐A84V, in which only the fungicidal activity of fluopyram and isofetamid was impaired. Docking studies showed that C‐A84 is positioned near the aliphatic linker of fluopyram,^15^ and substitution of alanine with bulky valine might, in comparison with other SDHIs, have the greatest impact on isofetamid binding, to which its carbonyl group was introduced in its aliphatic spacer (Fig. 1). Considering the SDHI sensitivity profiles and similarity between chemical 3D structures, a similar mode of binding can be expected between fluopyram and isofetamid.

### 4.2 Z. tritici strains with reduced sensitivity to fluopyram and isofetamid

Field isolates with reduced sensitivity to SDHIs commonly used in cereals, such as bixafen, isopyrazam, penthiopyrad and fluxapyroxad, have recently been identified. Most field isolates carry single key amino acid substitutions in at least one *Sdh* subunit, sometimes in combination with other alterations that do not form part of the binding pocket and can also be detected in resistant strains. For example, C‐T79 N can be found alone or in combination with C‐I29V or with both C‐N33 T and C‐N34 T (Fraaije, unpublished). Strains carrying C‐N33 T and C‐N34 T, including reference strain MM20, are sensitive to SDHIs and have been reported previously.^23^, ^38^ Multiple key target‐site alterations have been found in lab mutants^14^ and in two field strains isolated in 2015 (Fraaije, unpublished results), but this might carry a greater fitness penalty. Fluopyram‐resistant field strains of *Z. tritici* have been detected previously, but have not been characterized further.^23^ In this study, we found a high number of field strains resistant to fluopyram and isofetamid, but no key *SdhB*, *SdhC* or *SdhD* alterations were detected (Table 4). Additional *SdhA* sequencing of a fluopyram‐ and isofetamid‐resistant strain Lyon 35 also revealed no mutations in comparison with the sensitive reference strain IPO323 (see NCBI XM_003857126.1). No common mutations found in 4 *Sdh* subunits among fluopyram‐ and isofetamid‐resistant strains led us to evaluate other possible mechanisms in fungicide resistance.

Target‐site overexpression has also been reported as a resistance mechanism in several fungi. Different evolutionary pathways, such as gene duplication^39^ and genetic alterations of transcription factors^40^ or promoter regions,^41^ resulting in constitutively or inducible *CYP51* overexpression have been linked with azole resistance. However, overexpression of Sdh genes as a resistance mechanism is unlikely because this would affect all SDHIs to some extent, and not only the fluopyram and isofetamid sensitivity.

ABC and MFS transporters are also involved in fungicide resistance. Upregulated *MgMFS1* by 519‐bp insertion in the promoter region of *Z. tritici* resulted in low to moderate resistance against chemically unrelated antifungal compounds.^28^ The presence of the 519‐bp *MgMFS1* promoter insert in strains Lyon 16 and NT321.17 correlated with resistance to both tolnaftate and fentin chloride, but the RF values for fluopyram and isofetamid were relatively low in these strains. The presence of highly fluopyram‐ and isofetamid‐resistant strains, tolnaftate sensitive and lacking the *MgMFS1* 519‐bp promoter insert, suggest that *MgMFS1* overexpression is not the driver for strongly reduced fluopyram and isofetamid sensitivity. Interestingly, fluopyram and isofetamid resistance was also observed in mitochondrial extracts of the corresponding fluopyram‐ and isofetamid‐resistant strains (Table 5). Some ABC transporters are located within mitochondria^42^ and further studies are needed to study their potential role in fluopyram and isofetamid resistance.

### 4.3 Evolution and practical impact of resistance against fluopyram and isofetamid

Under laboratory conditions, artificial mutagenesis is considered to be a powerful tool to detect possible mutations and predict the future evolution of resistance in fields.^43^ Mutagenesis studies of *Z. tritici* under selection by different SDHIs have shown that some mutations can confer different levels of resistance to different SDHIs, although some mutations conferred high resistance levels to all SDHIs tested.^14^, ^15^ The detection of strains highly resistant to fluopyram, and in particular isofetamid, in multiple locations as early as 2003 (Table 7), before the widespread introduction of a newer generation of SDHIs into the cereal market in 2010, indicates pre‐existing non‐target site resistance. We have also seen that European *Z. tritici* populations have developed acquired resistance only through a range of different Sdh target‐site mutations since 2012.^16^ In addition, strains with altered efflux pump activity, including *MgMFS1*‐overexpressing strains have recently spread in Europe as a response to selection by QoI, azole and SDHI fungicides. Highly isofetamid‐resistant strains (EC_50_ > 5.0 mg L^−1^) seem to be accumulating in populations sampled at Rothamsted over time. This accumulation is not due to the selection of strains carrying Sdh mutations because only a few strains with Sdh mutations were detected in 2016 (C‐T79 N, *n* = 1) and 2017 (C‐T79 N, *n* = 2 and C‐R151M, *n* = 1), and all field Sdh variants reported to date are sensitive or slightly resistant to isofetamid (EC_50_ < 5.0 mg L^−1^). Highly isofetamid‐resistant strains might be selected indirectly through *in planta* breakdown products of SDHIs caused by metabolic activity of the host plant and/or the fungus itself. The high frequency of low isofetamid‐ and bixafen‐resistance strains in the 2017 Irish population can be explained in a sharp increase in frequency of efflux pump‐overexpressing strains and C‐T79 N strains after 2015.^44^ This study shows that non‐target site SDHI resistance pre‐exists in *Z. tritici* populations and should be considered for the development of new molecules and rational design of resistance management strategies.
